# Genetic and Environmental Associations Between Depressive Symptomatology and Loneliness in Adulthood

**DOI:** 10.1007/s10519-026-10280-2

**Published:** 2026-08-25

**Authors:** Morgan E. Lynch, Molly Gonenne, Colin D. Freilich, Thalida Em Arpawong, Deborah Finkel, Eric Turkheimer, Christopher R. Beam, Deborah W. Davis

**Affiliations:** 1 University of Southern California, Los Angeles, USA; 2 University of Minnesota - Twin Cities , Minneapolis , USA; 3 University of Virginia , Charlottesville, USA; 4 University of Louisville , Louisville, USA; 5 Jönköping University , Jönköping, Sweden

**Keywords:** Loneliness, Depressive symptomatology, Behavior genetics, Adulthood

## Abstract

The conceptual and symptom overlap between depression and loneliness is so strong that depressive symptom rating scales often include measures of loneliness, and their linear association ranges between .40 and .68. Despite these findings, few genetically informed studies have investigated the genetic and environmental association between them. We tested the degree to which depressive symptomatology and loneliness are correlated genetically and environmentally, and whether the environmental correlation in men is larger than in women. In this study, we used 428 individual twins (229 MZ twins and 187 DZ twins) from 266 families (139 MZ families and 127 DZ families) from the Louisville Twin Study (M_Age_ = 48.17; 56% female). Loneliness was measured using the UCLA Loneliness Scale (3 items), and depressive symptomatology was measured using the Center for iologic Studies Depression Scale. Statistical analysis included twin correlations and a correlated factors model. Results indicate that the genetic correlation between loneliness and depressive symptomatology is .47 [.95CI: .16–.78] and the nonshared environmental correlation is .28 [.95CI: .12–.43]. No sex differences were observed. In mid-adulthood, a little less than half of the genetic variance associated with depressive symptomatology is also associated with loneliness. Nonshared environmental factors underlying depressive symptomatology account for approximately 54.4% of the strong association with loneliness, too. These results are similar to bivariate estimates reported in a previous studies of young adulthood, suggesting that across adulthood, the contribution of genetic and non-shared environmental factors to the association between loneliness and depression is constant.

Loneliness (or perceived social isolation) and depressive symptomatology (i.e., symptoms consistent with clinical depression characterized by anhedonia and dysphoric mood) are relatively common and highly comorbid yet distinct phenomena across the lifespan (Lee et al. 2021). Epidemiological evidence in adulthood estimates the prevalence of loneliness to be 28.5% (Chawla et al. 2021) and 27.0% for depressive symptomatology (Wang et al. 2017). The impact of these psychological phenomena on individuals’ lives is substantial. Lonelier and more depressed individuals have disproportionate difficulty completing activities of daily living, experience less physical mobility, and have an increased risk for morbidity and mortality as they age (Lenze et al. 2005; Perissinotto et al. 2012).

Loneliness and depressive symptomatology (hereafter *depression* for simplicity) are frequently characterized by helplessness and emotional pain, both of which may be influenced by traits that are genetically and environmentally influenced (e.g., neuroticism, rejection sensitivity; Vanhalst et al. 2012; Grocott et al. 2024). Because of these shared symptoms and traits, moderate to strong correlations between loneliness and depression have been reported across the lifespan (*r* = .40-.68; Chang 2018; Chen et al. 2023). Both loneliness and depression are highly polygenic, which may contribute to their strong correlation. In a genome-wide association study of loneliness in older adults, the genetic correlation between loneliness and depression symptoms was moderate (*r*_g_=.39), and Mendelian Randomization (MR) studies support a bidirectional causal relationship between loneliness and depression (Sbarra et al. 2023; Sun et al. 2026). In a study of the polygenic overlap between loneliness and multiple psychiatric disorders, loneliness most strongly overlapped with major depression (*r*_g_ = 0.54; Rødevand et al. 2021), a similar MR study found loneliness was genetically most correlated with a latent factor encompassing neurodevelopmental and mood disorders (Martin et al. 2023). Similar environmental exposures also contribute to the development of these phenotypes. For example, stressful life events, including childhood maltreatment, and changes in interpersonal relationships such as divorce, are common environmental exposures underlying both phenotypes (Saveanu and Nemeroff 2012; Matthews et al. 2022; Kretschmer et al. 2018). We, thus, reasonably expect them to have a common genetic and environmental etiology across the life-span.

Only one behavior genetic study to date has quantified the genetic and environmental overlap between loneliness and depression in young adults, with evidence for a strong genetic correlation (*r*_G_ = .63) and small non-shared environmental correlation (*r*_E_ = .26; Matthews et al. 2016). However, replication in other samples of young adults and in other age groups has not been conducted. Loneliness and depression each have a U-shaped pattern across the lifespan, with increases in young and older adulthood and a nadir in mid-adulthood (Graham et al. 2024; Sutin et al. 2013). Although previous research suggests relative stability in the genetic and environmental contributions to loneliness across childhood to young adulthood (Boomsma et al. 2005), the environmental conditions across mid-to-late life warrant further investigation. Changes in people’s interpersonal relationships, such as divorce or the death of a spouse, as well as changes in functional ability with age, underlie some of the increase in loneliness and depression in adulthood (Zhai et al. 2024; Pearlin and Johnson 1977; Barjaková et al. 2023; Hawkley et al. 2022; Pollack et al., 2023; Yang and George 2005). Given these situational and social differences across the lifespan, genetic and environmental contributions to both loneliness and depression may differ between young adults and older adults.

Additionally, prevalence and etiology across the lifespan may be moderated by sex. Evidence on sex differences in loneliness is inconsistent, with studies variously reporting no between-group differences (Maes et al. 2019), higher loneliness among women (Nicolaisen & Thoresen, 2024; Pinquart & Sorenson, 2003), and higher loneliness among men (Borys and Perlman 1985). Given hypothesized social and cultural influences on loneliness (American Psychological Association, 2025), the environmental risk factors and maintaining mechanisms of loneliness may differ between men and women. Male participants’ fears of social judgment have been found to inhibit their self-report of loneliness and help-seeking (Borys and Perlman 1985; Cramer and Neyedley 1998); consistent with this, studies using indirect measures (e.g., the UCLA Loneliness Scale; Russell et al., 1996) have shown higher levels of loneliness among men (Borys and Perlman 1985). Across men and women, common predictors are widowhood, depressive symptoms, and early-life adversity (Nicolaisen and Thorsen 2024). However, women’s self-reported loneliness is more often associated with partnership transitions (e.g., divorce, re-partnering) and physical health limitations, whereas being unpartnered and having fewer close social ties beyond an intimate partner are associated with men’s reports of loneliness (Nicolaisen and Thorsen 2024).

The etiology of depression also may differ by sex. Women report higher levels of depression across all ages, although sex differences are greatest in adolescence (Kessler et al., 2003; Salk et al., 2017). Women’s depression is more strongly associated with interpersonal and emotional factors (e.g., parental warmth, neuroticism, divorce, and marital satisfaction), whereas men’s is more strongly associated with exposure to external stressors and adversity (e.g., childhood sexual abuse, drug abuse, and financial, occupational, and legal life events) (Kendler and Gardner 2014). Biologically, stress reactivity and neuroendocrine functioning may also contribute to higher prevalence of depression rates in women compared to men (Nolen-Hoeksema, 2006). Together, different life experiences and environments, as well as biological differences in stress reactivity, may result in differences in men’s and women’s experience of loneliness and depression. Thus, the genetic and environmental associations between loneliness and depression may also differ by developmental epoch and sex.

Personality and social factors are known to correlate with loneliness and depression, both of which may confound estimation of their genetic and environmental association. First, high neuroticism has been found to account for variance in loneliness (Freilich et al. 2023; Buecker et al. 2020), an association that also has been found to be genetic in nature (Abdellaoui et al. 2019). Similarly, neuroticism and depressive symptoms are strongly correlated (Gupta et al. 2024). Second, social factors, like interpersonal relationships, also may confound the association between loneliness and depression (Zhang and Dong 2022; Billings et al. 1983; Kawachi and Berkman 2001). Marriage, for example, is associated with lower loneliness (Stack 1998) and depressive symptoms (Frech and Williams 2007) whereas being divorced, widowed, or single increases risk for both (Barjaková et al. 2023; Hawkley et al. 2022; Zhai et al. 2024; Pearlin and Johnson 1977). As a result of these associations, in the current study we adjust for effects of neuroticism and marital status on loneliness and depressive symptoms so that reported genetic and environmental correlations are unconfounded by these personality and social characteristics.

A clear understanding of the unbiased association between loneliness and depression may be helpful to researchers and clinicians alike to inform diagnostic properties of depression and interventions. If covariation is more genetically driven, we would expect to see stable, strong correlations across the lifespan. However, if covariation is driven more by environmental factors, we would expect their correlation to be variable over the life-course as various circumstances and exposures occur. Further explication of the common genetic and environmental contributions to loneliness and depression may help to inform theoretical models of loneliness and depression as symptoms that share common etiologies rather than causally related experiences (Cacioppo et al. 2006, 2010; Chen et al. 2023).

The aims of this paper are two-fold. We first quantify the genetic and environmental factors that account for the association between loneliness and depression in mid-life. We then test whether these associations differ by sex. We hypothesized that loneliness and depression would have a significant genetic and non-shared environmental correlation in mid-life, as previously found in young adulthood (Matthews et al. 2016). Given the age range and life-stage of our sample, we predicted that the genetic correlation would be stronger in the current study than Matthews et al.’s (2016) sample of young adults due to the stability of both during middle adulthood (Graham et al. 2024; Sutin et al. 2013). As an exploratory analysis (due to sample size limitations), we also hypothesized that genetic and environmental covariance between loneliness and depression would differ by sex, such that the environmental correlation for men would be greater than for women. The basis for this hypothesis is that men are more susceptible to environmental factors (e.g., perceived judgment, external stressors) in mid-to-late life that may affect their experience of loneliness and depression (Kendler and Gardner 2014; Cudjoe et al. 2020).

Three methodological considerations were addressed in the present study. First, the conceptual overlap between depression and loneliness is such that loneliness is itself an item indicator on one of the most common symptomatology scales, the Center for Epidemiologic Studies Depression Scale (CES-D; Radloff 1977). The loneliness item from each individual’s CES-D score was removed to improve the distinction between loneliness and depression. Second, to limit genetic confounding between our variables of interest, we controlled for neuroticism. Given the strong phenotypic and genetic associations between neuroticism, loneliness, and depression (Freilich et al. 2023; Jylhä and Isometsä 2006; Abdellaoui et al. 2019; Gupta et al. 2024), we adjusted for the associations that depression and loneliness have with neuroticism. Third, because consistent evidence that marital status is substantially associated with depressive symptoms (Zhai et al. 2024; Pearlin and Johnson 1977) and loneliness (Barjaková et al. 2023; Hawkley et al. 2022), we adjusted for effects of marital status on loneliness and depression before estimating genetic and environmental correlations between them.

## Method

### Participants

The current sample was drawn from the midlife study (2020–2024) of twins in the Louisville Twin Study (LTS), which is the first follow-up of the twins since they were 15 years old (Beam et al. 2020). Data were collected in Louisville, Kentucky. The twins were administered a neuropsychological assessment and a self-reported psychosocial assessment. The only exclusion criterion that we used was whether both twins did not provide data for either loneliness or depressive symptomatology. The analytic sample consists of 428 individual twins from 266 families. There are 139 MZ families: 94 complete pairs of twins and 45 incomplete pairs. There are 127 DZ families: 68 complete pairs of twins and 59 incomplete pairs. Among the MZ families, there are 58 complete and 24 incomplete MZ female pairs, and 36 complete and 21 incomplete MZ male pairs. Among the DZ families, there are 20 complete and 17 incomplete DZ female pairs, 19 complete and 20 incomplete DZ male pairs, and 29 complete and 22 incomplete DZ opposite-sex pairs. The current study was approved by the Institutional Review Board at the University of Louisville (19.0989) and the University of Southern California (UP-20–00029).

### Measures

*Depressive symptomatology* was measured using the 20-item Center for Epidemiologic Studies Depression Scale (CES-D; Radloff 1977). All twins completed the questionnaire prior to or during their in-person cognitive and clinical assessment visit. Twins rated how much they experienced each symptom over the past week on a 4-point Likert scale (0 = “Rarely or none of the time (less than 1 day)”; 1 = “Some or a little of the time (1–2 days)”; 2 = “Occasionally or a moderate amount of time (3–4 days)”; 3 = “Most or all of the time (5–7 days)”). All items were scored so that higher scores indicate worse symptomatology. One item within the CES-D that measures loneliness (“I feel lonely.”) was removed from the current study because of its overlap with our separately measured loneliness construct. Total scores were constructed by summing across the 19 items (possible scores range from 0 to 57). Scale reliability was substantial (omega = .92; Raykov & Shrout, 2002). Scores were log-transformed for homoscedasticity.

*Loneliness* was measured using the 3-item version of the UCLA Loneliness Scale (Russell et al., 1996). Twins rated the following items: “I feel that I lack companionship,” “I feel left out,” and “I feel isolated from others,” on a 4-point Likert scale (1 = “Always”; 2 = “Sometimes”; 3 = “Rarely”; 4 = “Never”). Scores were reverse-coded so that higher scores indicate greater feelings of loneliness. Total scores were constructed by summing across the 3 items (possibly scores range from 3 to 12). Scale reliability was substantial (omega = .89). Loneliness total scores were log-transformed for homoscedasticity.

The following covariates were included to control for potential confounds in the constructs of loneliness and depression: age, sex, marital status, education, and neuroticism. Age was a continuous variable indicating the participant’s chronological age at the time of participation. Education was coded as a ternary nominal variable: individuals who completed less than a Bachelor’s degree, individuals who completed a Bachelor’s degree, and those who completed more than a Bachelor’s degree. Marital status was coded as a binary nominal variable: individuals who were married and individuals who were not married, regardless of status (i.e., single, divorced, widowed). Neuroticism was measured with four items (“Have frequent mood swings.”; “Am relaxed most of the time.”; “Get upset easily.”; “Seldom feel blue.”) using a 7-point Likert scale (“Strongly Agree” = 1 to “Strongly Disagree” = 7) (McDonald’s omega = .64). Items were scored so that higher values indicate higher levels of neuroticism. Total scores were constructed by summing the four items.

### Data Analysis

Descriptive statistics for loneliness and depression, including phenotypic correlations across all twins, univariate twin correlations, and the cross-twin, cross-trait twin correlations are presented first. Univariate twin correlations are useful for indicating the presence (or absence) of additive genetic, shared environmental, and nonshared environmental variance underlying each construct. Greater MZ than DZ twin correlations suggest underlying additive genetic variance, and MZ twin correlations that are less than twice that of DZ correlations suggest shared environmental variance. Imperfect MZ twin correlations suggest the presence of nonshared environmental variance. Using the same pattern of differences between MZ and DZ twin correlations, cross-twin, cross-trait correlations show the presence of additive genetic, shared environmental, and nonshared environmental factors underlying the covariance between loneliness and depression.

**Fig. 1:**
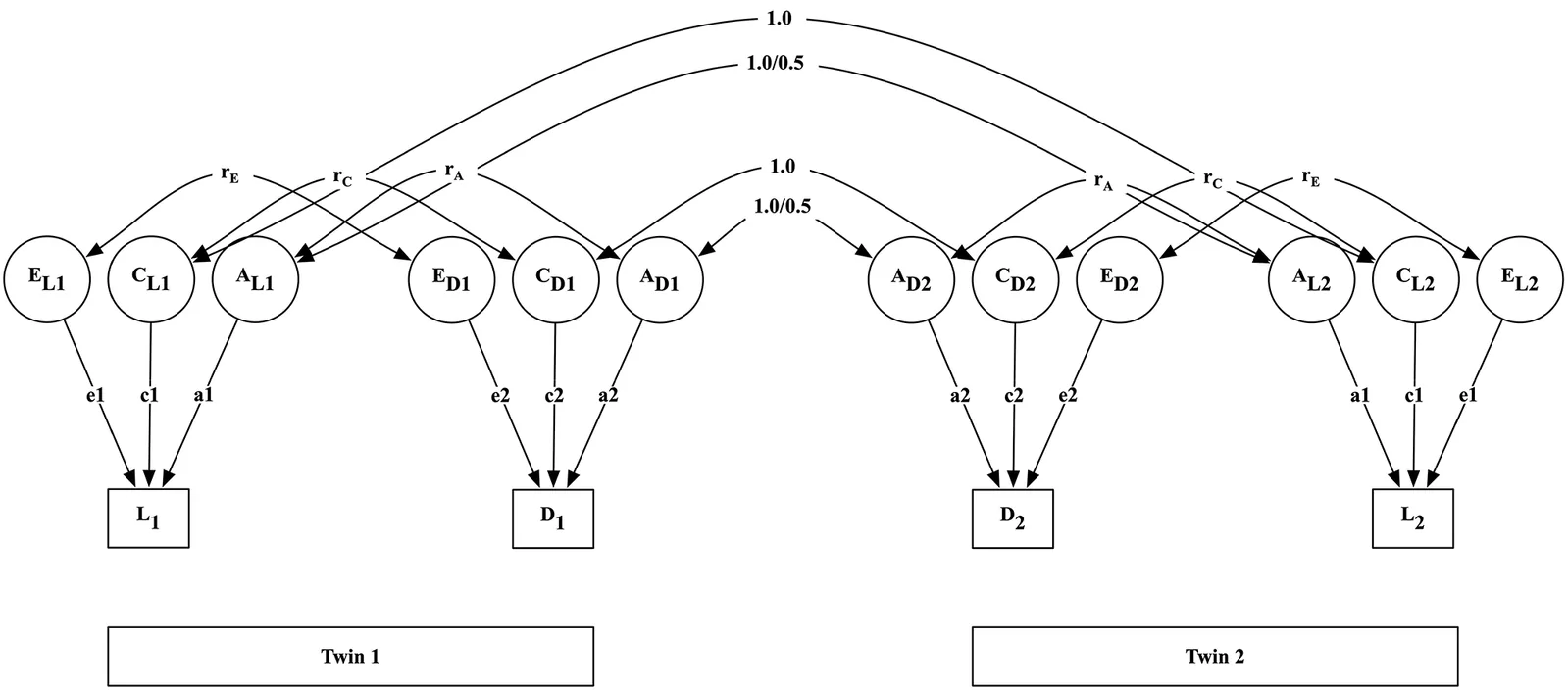
Correlated ACE Factors Model. L = loneliness; D = depression; A = additive genetic variance component; C = shared environmental variance component; E = nonshared environmental variance component; a = additive genetic effect; c = shared environmental effect; e = nonshared environmental effect. 1 = twin 1; 2 = twin 2

Multivariate model fitting consisted of correlated ACE factor models to estimate the genetic and environmental variance underlying loneliness and depression and their covariance (Fig. 1). The classical twin model makes a set of assumptions to identify the additive genetic, shared environmental, and nonshared environmental (co)variances between loneliness and depression. First, MZ twins share the same genotype whereas DZ twins share, on average, 50% of their segregating genes. Thus, the correlation between twins’ additive genetic variance components (A) for loneliness and depression, respectively, is 1.0 in MZ twins and 0.5 in DZ twins. Second, shared environmental variance components (C) contribute to the similarity between twin 1 and twin 2, irrespective of zygosity, and so are correlated 1.0 in both groups. Third, nonshared environmental variance components (E) comprise any factor, including measurement error, that make MZ twins different from one another, so these components are uncorrelated between twins. In addition to these three assumptions, genetic and environmental variance components for a given phenotype are uncorrelated and do not interact with one another. Lastly, non-assortative (i.e., random) mating is assumed to meet the assumption that DZ twins only share 50% of their segregating genes, on average.

To estimate the sources of covariation between traits, bivariate genetic and environmental covariation is decomposed into genetic and environmental components. The covariances between the additive genetic components of loneliness and depression are estimated, so that the genetic correlation (*r*_G_; Formula 1) can be estimated. In the same way, shared environmental correlations (*r*_C_; Formula 2), and nonshared environmental correlations (*r*_E_; Formula 3) are estimated using the covariances and variances of their respective components. Additionally, the proportion of the phenotypic covariance accounted for by genetic (*h*^2^) and environmental covariance (*e*^2^) is computed.

1 $${r_G}=\frac{{{\sigma _{{A_{CESD}}{A_{UCLA}}}}}}{{\sqrt {\left( {{\sigma ^2}_{{{A_{CESD}}}}*{\sigma ^2}_{{{A_{UCLA}}}}} \right)} }}$$

2 $${r_C}=\frac{{{\sigma _{{C_{CESD}}{C_{UCLA}}}}}}{{\sqrt {\left( {{\sigma ^2}_{{{C_{CESD}}}}*{\sigma ^2}_{{{C_{UCLA}}}}} \right)} }}$$

3 $${r_E}=\frac{{{\sigma _{{E_{CESD}}{E_{UCLA}}}}}}{{\sqrt {\left( {{\sigma ^2}_{{{E_{CESD}}}}*{\sigma ^2}_{{{E_{UCLA}}}}} \right)} }}$$

Parameter estimates were estimated separately for men and women using a five-group model (MZ males, MZ females, DZ males, DZ females, and opposite-sex DZ pairs). Two models were fit to the data, one that allowed estimates to vary between men and women and a second that constrained estimates to be the same across sex. These two models were compared using a chi-square difference test of nested models, the root mean square error of approximation (to test absolute model fit), and the Tucker-Lewis Index (to test comparative fit). All models were fitted in M*plus* 8.11 (Muthén & Muthén, 1998-2017) using robust full-information maximum likelihood (MLR) to adjust for missing data and the relatively modest sample size in the LTS. Full information maximum likelihood also allowed singletons to be included in the analysis. We included age, education, marital status, and neuroticism as covariates in both models.

## Results

### Descriptive Statistics

The average age of the sample was 48.17 and 56% of the sample was female (Table 1). In terms of race and ethnicity, the sample was 91% White and 6% Black and 2% multiracial and multi-ethnic. Across education levels, 43% of the sample had education less than a Bachelor’s degree (e.g., high school diploma, GED, unfinished Bachelor’s), 32% had a Bachelor’s degree, and 24% had completed more than a Bachelor’s degree. The average loneliness (*M*_UCLA_ = 5.34, *SD*_UCLA_ = 2.35) and depression (*M*_CES−D_ = 9.43, *SD*_CES−D_ = 9.20) scores were low to moderate in the sample, and the bivariate correlation between loneliness and depression was strong (*r* =.60).

**Table 1:** Descriptive statistics

	All Twins (*n* = 416)			MZ (*n* = 229)			DZ (*n* = 187)		
	*M* (%)	*SD*	Range	*M* (%)	*SD*	Range	*M* (%)	*SD*	Range
Age (years)	48.17	9.56	30–65	49.34	9.36	30–63	46.73	9.63	30–65
Loneliness (UCLA)	5.35	2.34	2–12	5.27	2.38	2–12	5.44	2.29	2–12
Depression (CES-D)	9.27	8.91	0–44	8.62	8.3	0–44	10.04	9.56	0–43
Neuroticism	13.71	4.25	4–28	13.75	4.29	4–28	13.66	4.20	4–26
Married	65%	-	-	67%	-	-	64%	-	-
Not married	35%	-	-	33%	-	-	36%	-	-
Female	56%	-	-	61%	-	-	50%	-	-
Education									
< Edu_BA_	43%	-	-	46%	-	-	41%	-	-
= Edu_BA_	32%	-	-	29%	-	-	36%	-	-
> Edu_BA_	24%	-	-	25%	-	-	23%	-	-
Race/Ethnicity									
White	92%	-	-	93%	-	-	90%	-	-
Black	6%	-	-	5%	-	-	7%	-	-
2 + Identities	1%	-	-	1%	-	-	2%	-	-

Edu_<BA_ refers to less than a completed Bachelor's degree, Edu_BA_ refers to a completed Bachelor’s degree, and Edu_> BA_ indicates education beyond the bachelor’s level. UCLA refers to the UCLA Loneliness Scale. CESD refers to the Center for Epidemiological Studies-Depression Scale

### Twin Correlations

Table 2 presents univariate and cross-twin, cross-trait correlations (CTCT). Univariate correlations were moderate for MZ twins (*r* =.41 for loneliness and *r* =.41 for depression); and small for DZ twins (*r* =.20 for loneliness, and *r* =.18 for depression). The univariate dizygotic twin correlations were considerably smaller than the univariate monozygotic twin correlations, suggesting the presence of additive genetic effects within-trait. In terms of CTCT correlations, loneliness and depression were correlated to a small degree across MZ twins (*r*s = .23–.33) and more weakly correlated across DZ twins (*r*s = .08–.24). In a model that constrained the CTCTs to be the same within zygosity group, the MZ loneliness-depression CTCT correlation was .27, whereas the DZ CTCT correlation was .16. Thus, the proportion of variance explained by the shared environment based on the constrained CTCTs was .05. Both sets of correlations suggest appreciable contributions from only additive genetic and nonshared environmental variance components.

**Table 2:** Twin Correlations

MZ	CESD_1_	LONE_1_	CESD_2_	LONE_2_
CESD_1_	1			
LONE_1_	0.51	1		
CESD_2_	0.41	0.23	1	
LONE_2_	0.33	0.41	0.61	1

DZ	CESD_1_	LONE_1_	CESD_2_	LONE_2_
CESD_1_	1			
LONE_1_	0.57	1		
CESD_2_	0.18	0.08	1	
LONE_2_	0.24	0.20	0.62	1

CES-D_1_ represents the total CES-D score for Twin 1. LONE_1_ represents the total UCLA-Loneliness score for Twin (1) CES-D_2_ represents the total CES-D score for Twin (2) LONE_2_ represents the total UCLA-Loneliness score for Twin 2. All scores were log-transformed

### Correlated ACE Factor Model Results

First, we compared an ACE model against an AE model and found that the shared environmental variances and covariance parameters could be eliminated from the model (∆^2^ = 0.165, *df* = 3, *p* =.961). Table 3 presents the unstandardized and standardized estimates of the correlated AE factor model. Additive genetic variance accounted for 30.7% of the variance in loneliness and 35.2% of the variance in CES-D. Adjusting for the effects of all covariates (sex, age, marital status, education, and neuroticism), the genetic correlation between loneliness and depression was strong (*r*_G_= .47, .95CI: .16–.78). Bivariate heritability was estimated to be 45.5% of the total phenotypic covariance. Nonshared environmental variance accounted for 69.3% of the variance in loneliness and 64.8% of the variance in CES-D. The nonshared environmental correlation between loneliness and depression was strong (*r*_E_ = .28, .95CI: .12–.43). Among the covariates, neuroticism, educational attainment, and marital status significantly predicted loneliness, whereas only neuroticism was associated with depressive symptomatology.

**Table 3:** Correlated AE factor model unstandardized (b) and Standardized (β) Estimates

	Parameter	b	95% CI	β	95% CI
Variances and Covariances					
	**A**_**CESD**_	**0.20**	**[0.10**,** 0.30]**	**.45**	**[.34**,** .56]**
	**E**_**CESD**_	**0.37**	**[0.28**,** 0.46]**	**.61**	**[.53**,** .68]**
	**A**_**UCLA**_	**0.04**	**[0.02 **,**0.06]**	**.20**	**[.14**,** .25]**
	**E**_**UCLA**_	**0.09**	**[0.07**,** 0.11]**	**.30**	**[.26**,** .33]**
	$\:{cov}_{{{A}_{UCLA,}A}_{CESD}}$	**0.04**	**[0.01**,** 0.07]**	**.47**	**[.16**,** .78]**
	$\:{cov}_{{{E}_{UCLA,}E}_{CESD}}$	**0.05**	**[0.02**,** 0.09]**	**.28**	**[.12**,** .43]**

Covariates					
	Age → CESD	−0.003	[−0.01, 0.01]	− .03	[−.12, .05]
	**Marital Status → CESD**	**−0.42**	**[−0.58**,** −0.26]**	**− .22**	**[−.30**,** − .14]**
	**Neuroticism → CESD**	**0.12**	**[0.11**,** 0.14]**	**.50**	**[.43**,** .58]**
	Sex → CESD	0.01	[−0.10, 0.13]	.00	[−.06, .07]
	Edu_BA_ → CESD	0.01	[−0.10, 0.13]	.00	[−.05, .06]
	Edu_> BA_ → > CESD	−0.01	[−0.07, 0.06]	− .01	[−.04, .03]
	Age → UCLA	0.001	[−0.002, 0.01]	.03	[−.06, .12]
	**Marital Status → UCLA**	**−0.26**	**[−0.31**,** −0.17]**	**− .30**	**[−.39**,** − .21]**
	**Neuroticism → UCLA**	**0.05**	**[0.04**,** 0.05]**	**.41**	**[.33**,** .49]**
	Sex → UCLA	−0.01	[−0.07, 0.07]	− .01	[−.09, .07]
	Edu_BA_ → UCLA	0.04	[−0.05, 0.11]	.04	[−.05, .12]
	Edu_> BA_ → > UCLA	0.001	[−0.11, 0.08]	.00	[−.09, .10]

Sex was coded such that female is the reference group, effects shown are the estimated effect of being male. Marital status was coded such that married is the reference group, effects shown are the estimated effect of being not married. Effects of education are coded such that the reference group are individuals with less than a Bachelor’s degree. Edu_BA_ refers to a completed Bachelor’s degree, and Edu_> BA_ indicates education beyond the bachelor’s level. UCLA refers to the UCLA Loneliness Scale. CESD refers to the Center for Epidemiological Studies-Depression Scale. Bolded values are significant at the 0.05 significance level

### Sex-Specific Twin Correlations and ACE Factor Model Results

For our exploratory analysis, we first present twin correlations by sex (Table 4). In the female MZ twins, the univariate twin correlation for loneliness was .45 and for depression was .40, and the average CTCT was .29. In the female DZ twins, the univariate twin correlation for loneliness was .30 and for depression was .37, and the average CTCT was .21. In the male MZ twins, the univariate twin correlation for loneliness was .34 and for depression was .43, and the average CTCT was .26. In the male DZ twins, the univariate twin correlation for loneliness was .14 and for depression was − .04, and the average CTCT was .04. In the opposite sex DZ twins, the univariate twin correlation for loneliness was .28 and for depression was .16, and the average CTCT was .24. When split by sex, twin correlations suggest additive genetic and nonshared environmental variance components underlying the association between loneliness and depression, with shared environmental variance contributing to the association in female twins.

Given the presence of shared environmental variance underlying loneliness, depression, and their association in the female twins, we tested shared environmental variance components in the female twins in a correlated ACE model and found that they could be constrained to zero without loss of model fit (Wald = 0.29, *df* = 3, *p* =.963). Next, we tested a 5-group sex limitation AE model. Model fitting results did not support evidence of sex differences in the genetic or nonshared environmental correlations (Wald = 0.45, *df* = 2, *p* =.797).

**Table 4:** Univariate twin correlations, cross-twin cross-trait correlations, and number of twin pairs by zygosity and sex

	*N*_pairs_	*r*_UCLA,CESD_	*r*_UCLA_	*r*_CESD_
MZF	82	0.29	0.45	0.40
DZF	37	0.21	0.30	0.37
MZM	57	0.26	**0.34**	**0.43**
DZM	39	0.04	0.14	− 0.04
DZOS	51	0.24	0.28	0.16

MZF = female MZ twins, DZF = female DZ twins, MZM = male MZ twins, DZM = male DZ twins, and DZOS = opposite sex DZ twins. Sample sizes (N) include both complete and incomplete pairs. Correlations presented are the average of the two estimates. UCLA refers to the UCLA Loneliness Scale. CESD refers to the Center for Epidemiological Studies-Depression Scale. Bolded values are significant at the 0.05 significance level

## Discussion

Although loneliness and depression are highly correlated phenotypically, no behavioral genetics study to date has investigated whether and to what degree they share common genetic and environmental etiologies in middle adulthood. Compared to one prior study in a young adult sample (Matthews et al. 2016), the genetic correlation in our sample of midlife adults is marginally smaller than the correlation found in young adults, whereas the non-shared environmental correlation is nearly identical (Matthews et al. 2016). Together, these results suggest that across young adulthood to mid-adulthood, the contribution of genetic and non-shared environmental factors to the association between loneliness and depression is relatively constant, irrespective of age-to-age differences in the prevalence of loneliness and depression across the lifespan (Graham et al. 2024; Sutin et al. 2013). However, more research is needed across the lifespan, specifically longitudinal research, to replicate these findings.

The strong genetic correlation observed in our study suggests that the genetic variation underlying depression underlies loneliness, too. Genetic correlations can arise for a number of reasons, first and foremost, because the genes associated with one disorder (here, depression) are associated with another characteristic (here, loneliness). Alternatively, genes underlying a third characteristic (e.g., neuroticism or rejection sensitivity) associated with both depression and loneliness also may explain genetic correlations (Gao et al. 2017). To illustrate this idea, we consider the experience of a twin going through a divorce in mid-adulthood. If one twin is divorced in mid-life and the other twin remains married, differences may arise between the twins’ feelings of both loneliness and depression for a number of different reasons. For instance, differences may emerge from the effects of the loss (Matthews et al. 2022; Kretschmer et al. 2018), their genetic propensity for neuroticism (Abdellaoui et al. 2019; Gupta et al. 2024)—a susceptibility to experience negative affect, cope maladaptively, and interpret any neutral information negatively—or the neurobiology of their stress response (Goossens et al. 2015; Tafet and Nemeroff 2016). In the present study, our bivariate estimates control for the confounding effects of neuroticism. The genetic correlation observed in the present study thus represents the unconfounded correlation between loneliness and depression due to unmeasured genetic factors. For example, prior research has found that genes important for several cellular processes, neurotransmitters, and the immune system are shared between loneliness and depression (Rødevand et al. 2021; Goossens et al. 2015; Tafet and Nemeroff 2016).

Just as the genetic correlation suggests that loneliness and depression share some of the same genetic factors, the nonshared environmental correlation observed in our study suggests that similar environmental factors, broadly construed, underlie loneliness and depression. It should be noted that the estimate of the non-shared environmental correlation includes all non-genetic relatedness between the measures, including correlated measurement error. The magnitude of the association is less than the genetic correlation, but large enough to suggest that environmental causes of depression, should they exist, are nontrivially associated with loneliness (and vice versa). Together, these results contribute to interpersonal theories of depression (IPT), which state that depressive symptoms are maintained by reciprocal relationships between intra-personal (e.g., cognitive and behavioral processes) and inter-personal interactions (Teichman and Teichman 1990). Continuing the example of divorce, IPT posits that the divorced twins’ loneliness and depression may be influenced not only by this negative interpersonal experience, but also maintained by their internal thoughts (e.g., negative beliefs about themself) and interpersonal behaviors (e.g., excessively seeking reassurance) (Coyne 1976). Often, people’s reactions to rejection or loss create a positive feedback loop for their perceived isolation and depression (Weissman 2006; Segrin 1996) because when the individual seeks excessive reassurance from others, and those conversations become increasingly self-focused, other people in the individual’s social network experience may negative affect and withdraw from social interactions (Coyne 1976). The present study supports the notion that people’s unique environments shape and maintain their loneliness and depression by similar mechanisms, such as through the cycle depicted in IPT.

The finding that overlapping nonshared environmental components explain the majority of the covariance between depression and loneliness has implications for future clinical research, specifically, the importance of quantifying loneliness in the context of treatments for depressive disorders. There are no evidence-based treatments for loneliness, and for good reason: loneliness is not a diagnosable psychiatric disorder but an acute (e.g., situation-dependent) or chronic (e.g., personality-dependent) set of conditions that are secondary (or even tertiary) symptoms of disorders like major depressive disorder and persistent depressive disorder. In cases where clients and patients report simultaneous symptoms of depression and endorse higher levels of loneliness, first-line treatments for depression designed to help people engage with features of their environments in more productive ways (e.g., behavioral activation, cognitive restructuring, interpersonal psychotherapy) have a greater chance of reducing feelings of loneliness, too (Lasgaard et al. 2026; Reinhard et al. 2021). Indeed, in a meta-analysis of interventions for loneliness, studies with the largest effect sizes for reducing loneliness (e.g., cognitive restructuring-based, acceptance-based, and social behavioral activation interventions) are also evidence-based treatments for depression (Coppola et al. 2025). Our study suggests that these effects may be reciprocal; interventions most effective for loneliness may also benefit depression. Psychotherapy is itself an environment, so the strong environmental association between depression and loneliness implies that loneliness is likely to be treated by deploying treatments for depression.

A second implication for future clinical research pertains to the finding that loneliness is a risk factor for depression (Cacioppo et al. 2006, 2010). The common genetic variance found here does not confirm or refute whether loneliness causes depression or whether people with a genetic predisposition for depression and loneliness report higher levels of loneliness and subsequently worse depressive symptoms, too. Here and elsewhere (Chen et al. 2023), findings lead us to believe that conclusions about the causal effects of loneliness on depressive symptoms are premature. Given the robust literature on treating depression, the logical step would be to tackle loneliness by way of treating depressive symptoms. In fact, under the belief that long-term psychotherapy is about addressing characterological problems (e.g., self-reported deficits in personality), evidence-based treatments for depression very well may interact with the common genetic variance underlying depression and loneliness to minimize people’s genetic expression of both.

A shortcoming of a behavior genetic study is that the latent genetic and environmental variables do not inform specific genetic or environmental features of people’s lives that are common to loneliness and depression. Genetic and environmental correlations, while useful for giving some sense of the overlap between genetic and environmental factors shared between depression and loneliness, do not clarify specific genetic or environmental causes or elucidate how they interact to explain their association. Moreover, the size of the correlations does not necessarily indicate which etiology accounts for the lion’s share of their covariance. The genetic and nonshared environmental covariances are nearly the same (0.04 and 0.05, respectively), reflecting less overall genetic relative to environmental variance in both loneliness and depression, but a higher degree of genetic relative to environmental overlap (i.e., correlation). Thus, our findings do not indicate that genes are somehow more important for explaining the phenotypic association between depression and loneliness. Indeed, nonshared environmental factors accounted for the greatest proportion of the phenotypic covariance (54.4%).

Several limitations of the current study are notable for the interpretation of results. First, we did not detect sex differences in twin bivariate twin correlations. The sample is relatively small, particularly for opposite-sex twins, so the sex-limitation analysis may be underpowered to detect differences between sexes. Future research should replicate these findings in a larger sample. Second, the observational design of the data prevents causal conclusions between loneliness and depression. Specifically, the genetic and environmental correlations observed between loneliness and depression do not imply that genetic or environmental factors underlying loneliness are causally related to depression. Third, the sample is relatively small for a twin study design and consists of more White participants than the general population of the United States. Thus, the sample may not be representative of all Americans, specifically, racial and ethnic minorities. Fourth, the limitations of classical twin modeling assumptions apply to this study. For example, genetic and environmental factors may interact, and people typically do not mate randomly, which may affect genetic and environmental variance estimates (Røysamb and Tambs 2016). Finally, the results here define only the broad ratio of genetic and nonshared environmental factors that influence loneliness and depression. Thus, further mixed-methods research is needed. Despite these limitations, the current study provides insight into the shared etiology of loneliness and depression in adulthood.

## Data Availability

Transparency and Openness: We report how we determined our sample size, all data exclusions, all manipulations, and all measures in the study. The authors assert that all procedures contributing to this work comply with the ethical standards of the relevant national and institutional committees on human experimentation and with the Helsinki Declaration of 1975, as revised in 2008. The study design, hypotheses, and analytic plan were not preregistered. Participants of this study did not agree for their data to be shared publicly, so supporting data are not currently available. Interested researchers, however, may consult Dr. Beam and Dr. Davis at beamc@usc.edu and deborah.davis@louisville.edu respectively, about potentially acquiring data used in the current study. All measures and Mplus analytic code used for the study are publicly available at: https://osf.io/j73dr/?view_only=5cbb2449db7040afbebe86793d0804ac.
